# Neurotheology: Practical Applications with Regard to Integrative Psychiatry

**DOI:** 10.1007/s11920-024-01584-3

**Published:** 2025-01-04

**Authors:** Andrew B. Newberg

**Affiliations:** Department of Integrative Medicine and Nutritional Sciences, Thomas Jefferson University, 925 Chestnut Street, Suite 120, Philadelphia, PA 19107 USA

**Keywords:** Religion, Spirituality, Mental health, Depression, Anxiety, Meditation, Prayer, Psychedelics

## Abstract

**Purpose of Review:**

Neurotheology is a nascent field of research and scholarship that seeks to understand the relationship between the brain and religious and spiritual phenomena. In the context of integrative psychiatry, neurotheology offers an intriguing intermediary between understanding how spirituality and religion affect brain function, and how this might be related to changes in mental health.

**Recent Findings:**

A number of research studies over the years have observed that religious and spiritual beliefs, practices, and experiences can have a profound impact on a person’s psyche. Many times, the effects are positive leading to lower depression, anxiety and distress. However, there are times that religion and spirituality can lead to negative beliefs and behaviors. Neurotheology seeks to understand both the positive and negative effects of religion and spirituality on mental health from a brain perspective. In addition, neurotheology offers important philosophical insights into the nature of the human mind and how we perceive the reality around us.

**Summary:**

This review evaluates these many topics as it considers how neurotheology can have practical applications with regard to integrative psychiatry.

## Introduction

Neurotheology is the field of study that seeks to understand the relationship between the brain and religious/spiritual phenomena [1]. As a field, its domain ranges from philosophical and theological questions to practical applications that may have important implications for the field of psychiatry. This review will broadly consider the aspects in the field of neurotheology that may be relevant in the context of integrative psychiatry. In particular, we will consider important philosophical topics in terms of understanding the human mind, and it’s normal and abnormal functioning. We will review current research regarding the relationship between religion/spirituality and mental health. And we will consider how various spiritual practices may be useful in the field of integrative psychiatry. The latter includes practices such as meditation and prayer, but also includes recent work in the field of psychedelic experiences, particularly those that elicit spiritual experiences. Overall, neurotheology may play a prominent role in the future development of integrative psychiatry, seeking to better support mental health in patients regardless of their religious or spiritual beliefs.

## Neurotheology Foundations and Integrative Psychiatry

Before going further into the practical applications of neurotheology, it is important to first more clearly define the field. As mentioned, its basic goal is to determine how the brain interweaves with religion and spirituality. However, there are aspects of this field of study that are important to mention for a better understanding of the entire approach. For neurotheology to be useful, as both a term and a field, it is important to define both sides as broadly as possible [2]. Hence, the “Neuro” side should include cognitive neuroscience, neuroimaging, psychiatry, consciousness studies, and even anthropology and sociology. The “Theology” side should include not only theology proper, but all of the ways human beings engage religion and spirituality. This includes religious and spiritual beliefs, practices, rituals, and experiences.

In prior work, I have argued that it is also important that neurotheology be considered a “two-way street” [1]. This means that neurotheology is not just a neuroscientific evaluation of religion, nor a religious evaluation of neuroscience. Neurotheology needs to be truly open to both sides so that the science is done as rigorously as possible, while also ensuring that the religious or spiritual elements are maintained with as much integrity as possible. The reason for this is that if we were to perform a scientific study in which we wanted to test whether doing the rosary prayer reduces anxiety, we need to carefully design an adequate study to ensure the data is accurate and the power and statistics provide meaningful results. But it is also important that the rosary prayer itself is done correctly and by people who believe in it and fully engage its practice. If we were to study a group of atheists who did not believe in the practice, and then find that it did not reduce anxiety, it would not be an adequate evaluation of that practice and its effects. Similarly, our research team has performed a number of studies on people performing various spiritual practices while undergoing brain scanning. However, if they are unable to perform the practice in the usual manner with the usual spiritual or religious fervor, then the brain scan results would not be a valid reflection of the practice.

An important point about neurotheology in the context of integrative psychiatry also applies to definitions. How should one define spirituality or religiousness? Of course, such definitions are different depending on the expertise of the person providing them. A group of psychiatrists will have a different definition of spirituality compared to cognitive neuroscientists, and especially theologians, sociologists, or anthropologists. In the context of psychiatry, there are other definitions which are important to assess such as mind, consciousness, and even soul. Particularly relevant in the realm of psychiatry is the definition, or determination, of normal and abnormal states. Some scholars have considered all religious behavior to be abnormal or delusional [3]. However, many people acknowledge that spirituality and religiousness can be an important and adaptive part of people’s lives. Prayer and meditation are frequently cited as the most commonly used coping mechanisms when people are confronted with various health problems or stressors.

Some practices can appear abnormal to some and completely normal to others. We previously performed a study of speaking in tongues that involves Pentecostal Christians making unusual vocal utterances that sound like language, but have no clear linguistic correlates [4]. A well-known clinical case in one of our hospitals occurred when an older woman, who had been admitted to the intensive care unit, was found to be in a trance-like state making unusual vocalizations. The medical team assumed that she was becoming psychotic and prepared to treat her with neuroleptic medication. Fortunately, before administration of the medication, several family members came in and informed the staff that the woman was merely speaking in tongues as part of her religious and cultural background. They indicated that the woman did this when she was confronted with significant stressors such as being in the intensive care unit.

Was speaking in tongues, for this woman, a normal or abnormal process? Similarly, we might ask whether a person who perceives themselves to receive information from God (e.g. hearing the voice of God) to be psychotic and delusional, or just a fervently religious individual. Finally, many individuals are religious, but some take religiousness to an extreme, such as nuns and priests who withdraw from what might be considered “normal” life activities [5]. They do not get married, do not have children, and often renounce material goods so that they can devote themselves to their religious beliefs. Again, does this represent normal or abnormal behavior? Neurotheology would suggest that research can help us understand these different circumstances by studying them systematically, evaluating the psychological, behavioral, and biological components of these phenomena. And perhaps, neurotheology can help us understand why people view reality in vastly different ways with some intensely religious and some not religious at all [6].

## Religion/Spirituality and Mental Health Benefits

A large number of research studies over the past 30 years has generally documented positive effects of religious and spiritual attitudes on mental health. For instance, Koenig et al. [7] conducted a comprehensive review of research on religion and mental health, finding that religiousness is correlated with lower levels of depression, anxiety, and substance abuse. Additionally, individuals who engage in regular religious practices report higher levels of life satisfaction and happiness compared to their non-religious counterparts [7]. These effects tend to be protective across the lifecycle, including in children and adolescence [8].

Religion can influence alcohol and substance use at various stages, including initiation, extent of use, impact on life, and the ability to quit and recover [9]. Attitudes towards alcohol and substance use differ widely among religions. Some religious groups strictly forbid the use of alcohol and substances [10], while others discourage excessive use without outright prohibition. Leigh et al. found that college students with higher spirituality scores smoked and binge-drank less frequently [11]. A study by Miller et al. in a nationally representative sample of adolescents showed that personal devotion and affiliation with fundamentalist denominations were negatively associated with alcohol and illicit drug use [12]. Several factors may explain these findings such as fear of violating religious principles that can deter substance use. Religions often admonish adherents about the dangers of alcohol and drugs [13]. Peer pressure within religious communities can encourage abstinence, and the absence of peer pressure to use substances further supports this. Interestingly, certain spiritual traditions permit alcohol use, incorporating wine into rituals, and some utilize psychoactive substances like peyote, ayahuasca, and hashish for spiritual purposes [14].

Religion also serves as a potent coping mechanism, particularly in times of stress and adversity. Pargament [15] posits that religious coping strategies, such as prayer, meditation, and spiritual reflection, help individuals find meaning and purpose in their struggles, promoting resilience. This process of finding meaning can transform negative experiences into opportunities for personal growth and spiritual development.

Moreover, religious doctrines often promote forgiveness, gratitude, and hope—qualities that are inherently beneficial to mental health. For example, a study by Toussaint et al. [16] found that individuals who practiced forgiveness experienced lower levels of depression and anxiety. A neurotheological perspective explores how processes such as forgiveness are associated with various brain functions that support emotions such as empathy (insula and limbic regions), and cognitive restructuring (temporal and frontal lobe) [17]. And frontal lobe activity can modulate negative emotional reactions based in limbic function. Similarly, gratitude, which is frequently encouraged in religious teachings, has been linked to improved mood and overall psychological well-being [18].

It is important to distinguish between organized religion and personal spirituality, although both are often intertwined. Spirituality, which can be defined as a sense of connection to something greater than oneself, also plays a crucial role in mental health. Studies have shown that spiritual experiences and practices, even outside the context of organized religion, can reduce stress and enhance emotional well-being. For example, mindfulness meditation, which has roots in various spiritual traditions, has been widely adopted in secular settings for its mental health benefits. Research indicates that mindfulness practice can reduce symptoms of anxiety and depression and improve overall emotional regulation [19]. This latter point is important as there are certainly many practices that can be engaged in even if one is an atheist or agnostic. Practices such as mindfulness or yoga can be done by anyone regardless of their spiritual beliefs. And other spiritual-like activities such as being in nature or creative outlets like music can be very valuable in improving mental health.

## Religion/Spirituality and Mental Health Detriments

While spirituality and religiousness can provide significant mental health benefits, there are contexts where religious engagement can have detrimental effects on mental health. Well-known examples include cults as well as terrorist activity. In both cases, deep religious beliefs lead individuals to destructive behaviors, both for themselves and others in society. Little is specifically known about the neurophysiological differences between those who follow religion toward positive ends versus negative ends. But such research might be crucial for understanding these beliefs and behaviors, and ultimately helping people towards healthier ones.

Cults, often characterized by extreme and manipulative religious ideologies, can have profound adverse effects on mental health. Cult leaders typically exert significant psychological control over their followers, employing tactics such as isolation, indoctrination, and abuse to maintain dominance and compliance. The mental health impacts of cult involvement can be severe and long-lasting. Techniques such as brainwashing, coercive persuasion, and thought reform are common, leading to cognitive dissonance and emotional distress among members [20]. This manipulation can result in a loss of personal identity, increased anxiety, depression, and even psychotic symptoms in some cases.

Cults often isolate their members from outside influences, including family and friends. This social isolation can lead to a loss of social support networks, further exacerbating mental health issues. We might hypothesize that areas of the brain involved in social interactions such as the precuneus, frontal lobe regions, and amygdala might be affected and/or co-opted for engaging problematic beliefs [21]. Members become heavily dependent on the cult for emotional and psychological support, creating a cycle of dependence that is difficult to break [22]. Moreover, the authoritarian structure of cults can foster an environment of fear and paranoia. Members are often subjected to constant surveillance and punishment for non-compliance, leading to chronic stress and anxiety [23].

Such psychological effects are likely mediated by fear reactions based on amygdala and autonomic nervous system activity. These neurological changes could lead to permanent neurological consequences. However, no research studies have evaluated specific brain changes associated with cult behaviors and effects. This could be an important area of future research, especially for helping find methods to counter cult influences and help those affected to recover more effectively.

Religious terrorism represents another extreme where religious ideology negatively impacts mental health. Individuals involved in or affected by religious terrorism experience a range of psychological traumas, from radicalization to post-terrorism stress. The process of radicalization, where individuals adopt extremist ideologies, is often accompanied by significant psychological, and neurological, changes [24, 25]. Radicalized individuals may experience cognitive rigidity, moral disengagement, and heightened aggression. This radicalization can lead to alienation from family and community, further increasing psychological distress [26].

Studies have indicated that individuals involved in terrorist activities often exhibit higher levels of psychological distress, including anxiety, depression, and PTSD [27]. The indoctrination process, which often involves dehumanizing others and justifying violence, can lead to severe moral and emotional conflicts, resulting in long-term psychological harm. The psychological impact of religious terrorism extends beyond the perpetrators to the victims and broader communities. Survivors of terrorist attacks frequently experience acute stress reactions, PTSD, depression, and anxiety disorders. The trauma from such events can have long-lasting effects on mental health, disrupting individuals' lives and well-being [28].

Empirical studies provide evidence for the negative mental health impacts of involvement in cults and religious terrorism. A study by Lalich and Tobias [29] on former cult members found that many individuals experienced symptoms of PTSD, depression, and anxiety long after leaving the cult. The study underscored the deep psychological scars left by cult involvement. Similarly, research on the psychological effects of terrorism indicates high rates of PTSD and depression among both direct victims and those indirectly affected by terrorist events. A meta-analysis by Brewin, Andrews, and Valentine found that exposure to terrorist attacks was associated with significant increases in PTSD prevalence, highlighting the severe mental health consequences of such events [30]. Again, while little is specifically known about the brain effects of terrorist or cult beliefs and behaviors, many of the underlying elements such as brain washing or PTSD have been studied. In addition, a crucial element might be in-group versus out-group conceptions that involve the amygdala, fusiform gyri, orbitofrontal cortex, and dorsal striatum [31]. It is possible that if future research can establish some of the brain responses, more effective approaches for redirecting people to more positive perspectives on religion that support empathy, compassion, and forgiveness might be developed.

## The Beneficial Effects of Spiritual Practices on Mental Health

In returning to more positive aspects of religion/spirituality on mental health, it is important to note that spiritual practices have been increasingly recognized for their beneficial effects on mental health, particularly in managing stress, anxiety, and depression. The interplay between spirituality and mental well-being is supported by a growing body of research indicating that spiritual practices can significantly enhance mental health outcomes. For example, spiritual practices, such as meditation, prayer, and mindfulness, have been shown to reduce stress levels effectively. Meditation, in particular, has been extensively studied for its stress-relieving properties, and its beneficial effects on anxiety and depression symptoms. A meta-analysis of 47 trials found that meditation programs can reduce psychological stress, with moderate evidence supporting improved anxiety, depression, and pain outcomes [32]. Many meditation practices, even when secularized, are based on an underlying spiritual tradition such as Buddhism or Hinduism, but can be helpful for spiritual and non-spiritual individuals.

Mindfulness-based stress reduction (MBSR) is another practice that has gained popularity for its effectiveness in reducing stress. A systematic review and meta-analysis involving 29 studies concluded that MBSR significantly reduces stress and improves mental health outcomes in both clinical and non-clinical populations [33] (Khoury et al., 2015). Furthermore, a number of studies have explored the impact of the MBSR program on neuronal function in the default mode network and particularly on frontal-limbic interactions that are involved with the modulation of emotional reactivity [34].

Anxiety disorders are among the most common mental health issues, affecting millions worldwide. Spiritual practices offer a non-pharmacological approach to managing anxiety. Prayer and meditation are commonly practiced spiritual activities that have been linked to reduced anxiety levels. For instance, several studies [35] found that participants who engaged in regular prayer and meditation experienced significant reductions in anxiety compared to those who did not engage in these practices. Further, such clinical effects may be mediated by structures such as the amygdala, caudate, prefrontal cortex, inferior frontal lobe, parietal region, and cingulate cortex [36].

Depression is a major mental health disorder that can severely impact an individual's quality of life. Spiritual practices, particularly those involving meditation and mindfulness, have shown promise in alleviating depressive symptoms. Mindfulness-based cognitive therapy (MBCT), which combines mindfulness practices with cognitive-behavioral techniques, has been found to be particularly effective. A randomized controlled trial by Kuyken et al. (2015) involving 424 participants with recurrent depression found that MBCT was as effective as maintenance antidepressant medication in preventing relapse. Other studies evaluating mantra based practices such as Transcendental Meditation have found mild to moderate improvements in depression, along with anxiety symptoms [37].

Interestingly, a study by Wachholtz and Pargament [38] compared the effects of secular meditation, spiritual meditation, and relaxation on patients with depression. The study found that participants in the spiritual meditation group, which included prayer, experienced greater reductions in depressive symptoms compared to the other groups. This finding indicates that prayer can enhance the effectiveness of traditional therapeutic approaches for depression.

In terms of the mechanism, as mentioned, a number of studies have found spiritual practices to affect areas involved with emotional regulation. In addition, several studies have found that spiritual practices affect neurotransmitter systems. A study from our group found that a one week spiritual retreat program had significant effects on the dopamine and serotonin systems [39]. In particular, the dopamine and serotonin reuptake sites were altered in a manner that might be consistent with the effects of antidepressant medications such as SSRIs. Several studies have found meditation practices to increase GABA in the brain which is known to have anti-anxiety effects [40].

## Psychedelic Effects on the Brain and Mental Health

Another intriguing area of exploration, that might also be considered from a neurotheological perspective, is the effects of psychedelic compounds on mental health. There is a substantial increase in research involving psychedelic compounds such as psilocybin on improving mental health conditions including depression and PTSD. Psilocybin has been shown to elicit spiritual-like experiences and such experiences seem to be associated with improvements in anxiety and depression symptoms [41]. Ongoing research is exploring how psychedelic compounds might be used clinically, perhaps in conjunction with psychotherapy. A benefit of studying psychedelic compounds is that their physiological mechanism is known, typically affecting the serotonin system. Recent neuroimaging studies have also evaluated the effect of these compounds on brain function and connectivity, particularly involving the limbic and striatal regions as well as the prefrontal cortex [42]. Additionally, psilocybin appears to affect connectivity in a number of brain networks including decreases in the default mode network and salience network, and increases within the central executive network [43].

## Conclusion

Neurotheology may play a fascinating role in future integrative psychiatry by helping to find the link between brain processes, spirituality, and mental health. From developing a philosophical/theological basis for understanding how spirituality can affect mental health to evaluating the clinical utility of religious/spiritual practices, neurotheology provides an important intersection for such scholarship. Importantly, neurotheology can help bridge the gap between profound religious/spiritual experiences and the human psyche and perhaps can even provide an intriguing new perspective on age old epistemological questions about how we as human beings perceive the reality around us.

## Key References

- Álvarez-Pérez Y, Rivero-Santana A, Perestelo-Pérez L, et al. Effectiveness of Mantra-Based Meditation on Mental Health: A Systematic Review and Meta-Analysis. Int J Environ Res Public Health. 2022;19(6):3380. Published 2022 Mar 13. 10.3390/ijerph19063380
  - This article provides an updated analysis of the effects of mantra based meditation on mental health measures such as anxiety and depression. It provides an updated approach showing the detail and robustness of data in this field.
- Stoliker D, Novelli L, Vollenweider FX, Egan GF, Preller KH, Razi A. Neural Mechanisms of Resting-State Networks and the Amygdala Underlying the Cognitive and Emotional Effects of Psilocybin. Biol Psychiatry. Published online January 6, 2024. 10.1016/j.biopsych.2024.01.002.
  - This article reveals recent findings on the neurophysiological effects of the psychedelic drug, psilocybin which has important implications for understanding the mechanism that underlies the spiritual nature of the experiences associated with it, and how it might be useful for mental health problems.
- Newberg A, Smith MC. Catholic Neurotheology. Wilmington, DE: Vernon Press, 2024.
  - This book explores wide ranging topics regarding how neurotheology can address a specific spiritual tradition while considering existing brain research and also how the brain-spiritual relationship can affect the human psyche.

## Data Availability

No datasets were generated or analysed during the current study.
